# Effects of New Special Formula Fertilizer on Potato Growth, Yield, and Fertilizer Utilization Efficiency

**DOI:** 10.3390/plants14040627

**Published:** 2025-02-19

**Authors:** Fulin Xu, Ajing Meng, Yi Liu, Jiangtao Li, Nan Wu

**Affiliations:** 1 School of Hydraulic and Civil Engineering, Ludong University, Yantai 264025, China; fulinxuu@126.com; 2 Institute of Agricultural Resources and Environment, Xinjiang Academy of Agricultural Sciences, Urumqi 830091, China; mengajing@xaas.ac.cn; 3 National Soil Quality Akesu Observation and Experiment Station/Akesu Brown Desert Soil Quality Xinjiang Field Scientific Observation and Research Station, Akesu 842304, China; 4 Comprehensive Experimental Station, Xinjiang Academy of Agricultural Sciences, Urumqi 830091, China; liuyun_5511@163.com (Y.L.); xjnkyljt@163.com (J.L.); 5 School of Resources and Environmental Engineering, Ludong University, Yantai 264025, China

**Keywords:** potato fertigation, specialized fertilizer formulation, precision fertilization technology, ‘Xisen 6’ variety, fertilization efficiency

## Abstract

This study addresses the low yield and fertilizer utilization efficiency of potatoes in the arid region of Xinjiang, Northwest China. The effect of a novel, fulvic acid-based specialized fertilizer for drip application on potato growth, development, yield, and fertilizer efficiency is investigated. The aim is to identify a suitable fertilizer formula for potato cultivation in Xinjiang and promote its demonstration and application, providing a theoretical basis for improving fertilizer efficiency and achieving stable, high yields in the region. The experiment was conducted with potato variety ‘Xisen 6’ using a field trial design, which included five treatments: no fertilizer (CK), conventional fertilizer (CF: N-P-K = 258-245-338 kg·ha^−1^), formulated fertilizer 1 (F1: 120 g·L^−1^ fulvic acid, N-P-K = 110-100-120 g·L^−1^), formulated fertilizer 2 (F2: 60 g·L^−1^ fulvic acid, N-P-K = 130-120-150 g·L^−1^), and formulated fertilizer 3 (F3: 30 g·L^−1^ fulvic acid, N-P-K = 170-150-130 g·L^−1^). Agronomic traits, gas exchange parameters of leaves during the tuber expansion stage, and yield components at harvest were measured, along with the fertilizer use efficiency for each treatment. Results show that F1, F2, and F3 improved the potato plant height, stem diameter, aboveground dry weight, SPAD value, stomatal conductance (Gs), and yield. Among these, F2 significantly enhanced plant height, stem diameter, aboveground dry weight, and the accumulation of relative chlorophyll content (SPAD value) during the tuber expansion stage, increased photosynthesis, and further improved yield and fertilizer efficiency, resulting in a yield increase of 121.29% and 34.6% compared to CK and CF, respectively. Therefore, formula fertilizer F2 is identified as the optimal fertilization strategy for potato cultivation in Xinjiang. Its application has been further extended in demonstration trials. Field demonstration results show that F2 significantly increased potato yield, with a 14.79% yield increase compared to CK, proving its effectiveness in replacing conventional fertilizers and enhancing production.

## 1. Introduction

The potato (*Solanum tuberosum* L.), a member of the Solanaceae family, is the fourth largest food crop globally. It is highly adaptable, requires minimal environmental conditions for cultivation, and is rich in nutrients, serving as both a staple food and forage crop. In China, potatoes hold significant strategic importance for economic development and food security [1,2,3]. As a nutrient-demanding crop, potatoes have a particularly high requirement for nitrogen (N), phosphorus (P), and potassium (K). The proper supply of these essential nutrients is critical for tuber growth, yield, and quality, as any nutrient deficiency can lead to significant yield reductions [4,5,6].

Xinjiang, located in an arid region with vast territory, large diurnal temperature variations, and a cool climate, provides natural conditions that are well suited for potato cultivation. According to the Xinjiang Statistical Yearbook, the potato planting area in Xinjiang reached 20,310 hectares in 2020, with a total production of 157,900 tons. In southern Xinjiang alone, the planting area covered 9070 hectares, making potatoes one of the region’s most important cultivated crops [7].

Since the 1980s, China has accounted for 32% of the global fertilizer usage, yet its fertilizer use efficiency remains low, ranging from 30% to 35%—which is significantly below that of developed countries [6,8]. Excessive fertilizer application is a widespread practice in potato cultivation, resulting in increased production costs, reduced economic returns, soil compaction, declining soil fertility, nitrogen and phosphorus surpluses, and potential food safety risks [9,10]. Among these, excessive nitrogen (N) application is particularly problematic, as it leads to nitrate leaching and greenhouse gas emissions while significantly reducing nitrogen use efficiency (NUE), thereby contributing to both environmental pollution and economic losses [8,11]. In pursuit of higher yields, farmers frequently overapply nitrogen; however, studies indicate that N application rates exceeding 250 kg·ha^−1^ provide no significant yield benefits [12]. As a nitrogen-intensive crop, potatoes exhibit relatively low nitrogen uptake efficiency, and excessive fertilization leads to nitrate accumulation in the root zone, exacerbating fertilizer waste and economic losses [13,14]. Therefore, optimizing fertilizer formulations and determining appropriate application rates are essential strategies for improving NUE, enhancing the sustainability of potato production, and maximizing economic profitability. Novel specialized fertilizers for potatoes, which primarily consist of small-molecule active organic substances such as fulvic acid, are designed based on the nutrient demand patterns during the peak growth periods of potatoes, fertilizer economic efficiency, and goals of reducing fertilizer use while enhancing productivity. These fertilizers are developed by determining the appropriate ratios of their components, applying the principles of water solubility, slow-release, and chelation enhancement technologies, resulting in high-concentration, highly active, multifunctional, and complete nutrient fertilizers. Fulvic acid (FA), a key ingredient, contains various active functional groups and exhibits strong cation exchange capacity, chelation ability, buffering capacity, adsorption ability, and catalytic effects. Its performance surpasses that of natural humic acid. FA can improve soil physical and chemical properties, enhance the efficacy of pesticides and fertilizers, stimulate crop growth, and increase stress resistance, making it an ideal choice for reducing fertilizer use while improving crop yields [15,16]. Research has shown that the application of fulvic acid-based fertilizers can significantly increase potato plant height, stem diameter, leaf SPAD values, and yield, while also increasing starch and vitamin C content and reducing the levels of reducing sugars [17]. Moreover, supplementing with fulvic acid soluble fertilizers on top of conventional fertilizers further boosts potato yield [18]. In addition, fulvic acid has been proven to significantly increase the yield of other crops such as corn, onion, safflower, and chili; improve nitrogen fertilizer efficiency; increase soil nitrate and ammonium nitrogen content; and promote plant growth and environmental stress tolerance [19,20,21,22]. Further studies indicate that fulvic acid fertilizers can improve the soil environment in the root zone, enhance crop quality, and convert into humic substances that are easily absorbed by plants, promoting growth and increasing stress resistance [23]. Therefore, the application of novel fertilizers and the reduction of traditional fertilizers are essential technical measures for improving potato quality and yield while enhancing soil conditions.

Given the goal of promoting crop growth and increasing yields, the development of potato-specific fertilizers containing fulvic acid holds great potential. However, despite the clear advantages of fulvic acid fertilizers in stimulating crop growth, novel specialized fertilizer formulas for potatoes have not yet been widely adopted among farmers. Currently, most research on potato drip irrigation fertilization, both domestically and internationally, focuses on the application of single nutrients, such as nitrogen, phosphorus, or potassium fertilizers [24]. In contrast, studies on novel specialized water-soluble fertilizers for potatoes in the Xinjiang region remain limited. Therefore, this study uses the potato variety ‘Xisen 6’ as the experimental material and applies different formulations of potato-specific fertilizers. The objectives of the study are (1) to investigate the effects of novel specialized water-soluble fertilizers on potato growth and photosynthetic parameters, (2) to evaluate their impact on potato yield, and (3) to explore their role in improving fertilizer use efficiency. The ultimate goal is to provide theoretical guidance and technical support for rational fertilization and high-yield, high-quality potato cultivation, promoting sustainable potato production.

## 2. Results

### 2.1. Effects of Different Fertilizer Treatments on Potato Plant Growth

**Figure 1:**
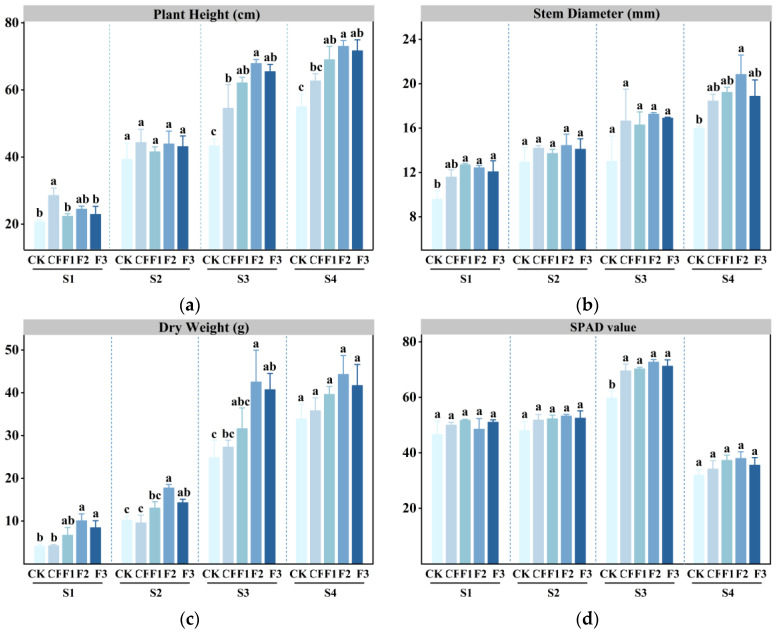
Effects of different fertilization treatments on plant height (**a**), stem diameter (**b**), above-ground dry weight (**c**), and relative content of chlorophyll (**d**) in potato at different growth stages. S1 = Seeding stage. S2 = Bud stage. S3 = Tuber swelling stage. S4 = Starch accumulation stage. The error bars represent the standard error of the mean (SEM). Different lowercase letters indicate significant differences among treatments (Tukey test, *p* < 0.05).

The plant height, stem diameter, and aboveground dry matter accumulation for each fertilization treatment gradually increased with the progression of the growth stages. The SPAD value showed a trend of initially rising and then decreasing throughout the growing period. As shown in Figure 1a, during the seedling stage, the plant height in the CF treatment was significantly higher than in the other treatments. During the tuber enlargement stage, the F2 treatment significantly promoted the plant height compared to both the CK and CF treatments. In the starch accumulation stage, the plant height under the F2 treatment reached its maximum. This was significantly higher than both the CF and CK treatments, increasing by 16.48% and 32.73%, respectively. Throughout the entire growth period, the potato plant growth followed a trend of initially rapid growth followed by a slowdown. As seen in Figure 1b, during the seedling stage, the absence of fertilization (CK) significantly reduced the stem diameter compared to the three fertilization treatments. In the starch accumulation stage, the stem diameter under the F2 treatment reached its maximum, significantly higher than the CK, but no significant difference was observed compared to the CF. The stem diameter increased by 13.02% and 30.19% over the CF and CK, respectively. In Figure 1c, it can be seen that during the seedling and bud stages, the aboveground dry matter of the F2 and F3 treatments was significantly higher than that of the CF and CK treatments. By the tuber bulking stage, the F2 treatment performed the best, significantly higher than both the CK and CF treatments. During the starch accumulation stage, the aboveground dry matter increased slowly, and the growth trend resembled that in the bulking stage, with the order of F2 > F3 > F1 > CF > CK. However, the differences between treatments were not significant. As shown in Figure 1d, except during the tuber enlargement stage, there were no significant differences in the relative chlorophyll content of potatoes treated with F1, F2, and F3 compared to the CK and CF treatments. However, during the tuber enlargement stage, the relative chlorophyll content reached its maximum. The relative chlorophyll content in the no fertilization (CK) group was significantly lower than in the CF, F1, F2, and F3 treatments, showing a 16.41%, 17.60%, 21.70%, and 19.24% decreased compared to the CF, F1, F2, and F3 treatments, respectively. No significant differences were observed between the F1, F2, F3, and CF treatments, indicating that adequate nutrient availability during the tuber enlargement stage significantly influenced the relative chlorophyll content.

### 2.2. Effects of Different Fertilizer Treatments on the Gas Exchange Parameters of Potato Leaves

**Figure 2:**
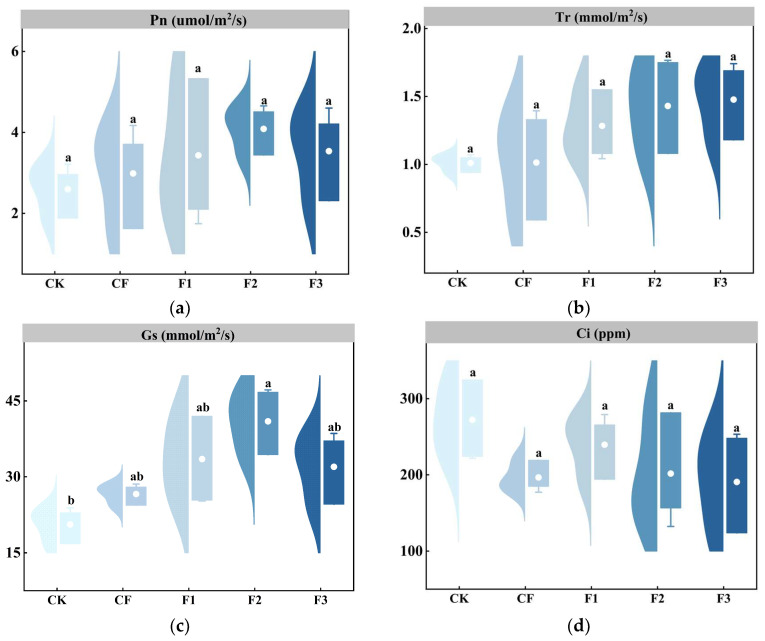
Effects of different fertilizer treatments on gas exchange parameters of potato leaves. (**a**) Pn = Net photosynthetic rate. (**b**) Tr = Transpiration rate. (**c**) Gs = Stomatal conductance. (**d**) Ci = Intercellular carbon dioxide concentration. The error bars represent the standard error of the mean (SEM). Different lowercase letters indicate significant differences among treatments (Tukey test, *p* < 0.05).

Different new specialized formula water-soluble fertilizers affect the gas exchange parameters of potato leaves, inducing responses in various photosynthetic traits. As shown in Figure 2a,b, while the net photosynthetic rate (Pn) and transpiration rate (Tr) under the fertilization treatments (F1, F2, and F3) were slightly higher than those under conventional fertilization (CF) and no fertilization (CK), no significant differences were observed between the treatments. In contrast, the stomatal conductance (Gs) values differed from the Pn and Tr, with the F2 treatment being significantly higher than CK, showing a 99.14% increase over CK. As indicated in Figure 2d, the trend in the intercellular CO_2_ concentration (Ci) was opposite to those of the Pn, Tr, and Gs, but no significant differences were found between the treatments.

### 2.3. Effects of Different Fertilizer Treatments on Potato Yield, Its Components, and Economic Benefits

**Figure 3:**
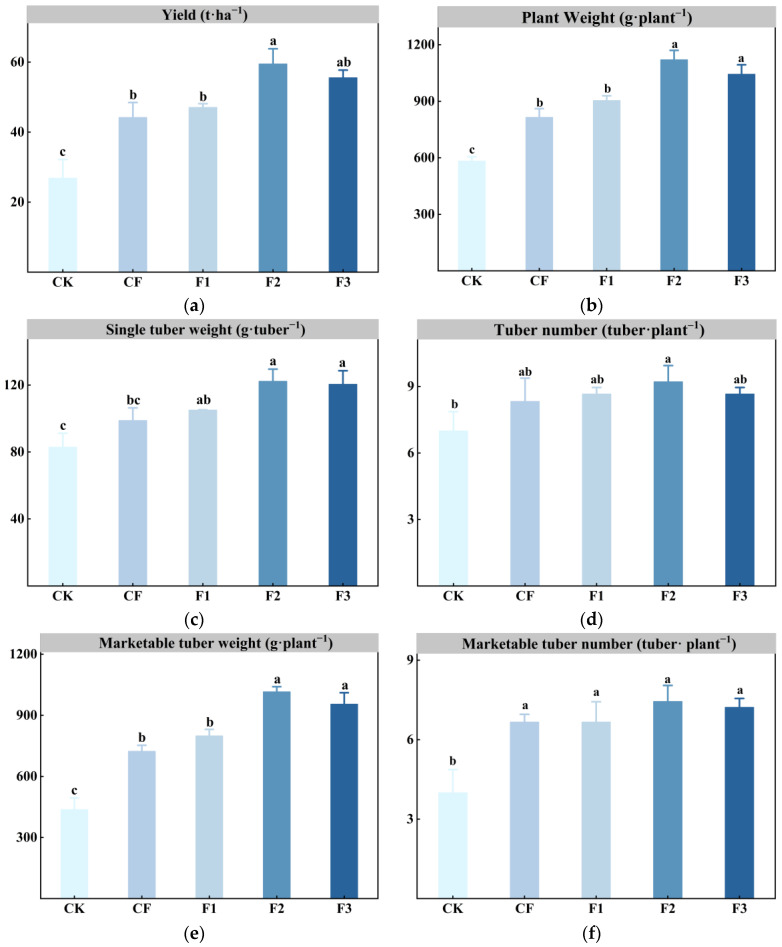
Effects of different fertilizer treatments on potato yield and its components. (**a**) Potato yield (t·ha^−1^). (**b**) Total tuber weight per plant (g·tuber^−1^). (**c**) Individual tuber weight (g·tuber^−1^). (**d**) Tuber number per plant (tuber·plant^−1^). (**e**) Marketable tuber weight per plant (g·plant^−1^). (**f**) Marketable tuber number per plant (tuber·plant^−1^). The error bars represent the standard error of the mean (SEM). Different lowercase letters indicate significant differences among treatments (Tukey test, *p* < 0.05).

Figure 3a shows the effect of different fertilization treatments on the potato yield. The results indicate that the F2 treatment significantly increased potato yield, reaching 59.49 t·ha^−1^, which was significantly higher than the CK, CF, and F1 treatments. Compared to the CK treatment (26.88 t·ha^−1^), the yield increased by 121.29%, and compared to the CF treatment (44.2 t·ha^−1^), it increased by 34.6%. The fertilizer usage in the F2 treatment was reduced by 29.41%, with the application rates of nitrogen (N), phosphorus (P), and potassium (K) decreasing by 28.39%, 29.60%, and 30.06%, respectively. This not only reduced the negative impact of the chemical fertilizers but also played a key role in saving fertilizer costs. The F3 treatment yielded 55.56 t·ha^−1^, showing a 106.70% increase over the CK treatment and a 25.70% increase over the CF treatment. Moreover, the trends in individual plant yield (Figure 3b), the individual tuber weight (Figure 3c), and the yield of marketable tubers per plant (Figure 3e) were consistent with the overall yield, with F2 > F3 > F1 > CF > CK. In summary, all three formula fertilizers increased potato yield, with F2 yielding the best results, followed by F3. The yield of F1 was comparable to that of CF, with no significant difference.

Table 1 shows that the economic benefit of the F2 treatment was the best, reaching 48,508.9 RMB·ha^−1^, representing an increase of 23,523.1 RMB·ha^−1^ compared to the CF. F3 followed with an economic benefit of 42,612.1 RMB·ha^−1^, an increase of 17,627.3 RMB·ha^−1^ over the CF, while F1 only contributed an increase of 4908.4 RMB·ha^−1^ over the CF. Overall, the F2 treatment had the highest total income, net income, increased income, and yield-to-investment ratio.

### 2.4. Effects of Different Fertilization Treatments on Fertilizer Use Efficiency in Potato

The fertilizer partial factor productivity (PFP) is a comprehensive indicator that measures the overall effect of fertilizer application [25]. As shown in Table 2, the fertilizer partial factor productivity values of the F1, F2, and F3 treatments were significantly higher than that of the CF treatment., while no significant difference was observed in fertilizer agronomic efficiency compared to the CF treatment. Among these, the F2 treatment had the highest PFP and AFUE, with values of 100.1 kg·kg^−1^ and 54.9 kg·kg^−1^, respectively, representing increases of 90.67% and 166.5% compared to the CF treatment. The F3 treatment ranked second, while the F1 treatment showed the lowest performance in terms of fertilizer efficiency.

### 2.5. Correlation and Principal Component Analysis of Potato Indicators Under Different Fertilization Treatments

**Figure 4:**
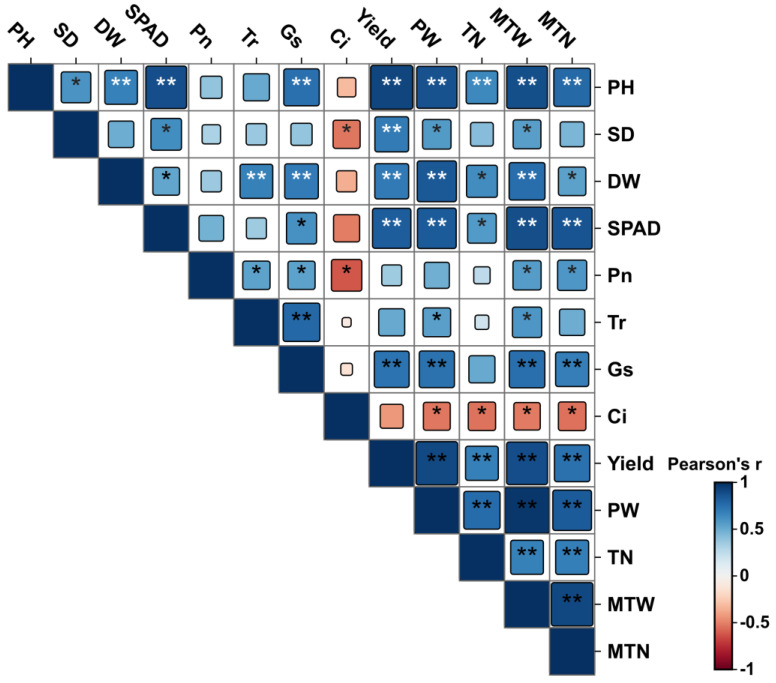
Correlation between potato growth parameters, photosynthetic indicators, yield, and its constituent factors. * and ** indicate significant differences at *p* < 0.05, and *p* < 0.01. PH = Plant height. SD = Stem diameter. DW = Aboveground dry weight. SPAD = Relative chlorophyll content. Pn = Net photosynthetic rate. Tr = Transpiration rate. Gs = Stomatal conductance. Ci = Intercellular carbon dioxide concentration. Yield = Yield per acre. PW = Plant weight. TN = Tuber number per plant. MTW = Marketable tuber weight. MTN = Marketable tuber number per plant.

As shown in Figure 4, through correlation analysis of agronomic traits, photosynthetic performance, aboveground dry matter accumulation, yield, and yield components during the tuber bulking stage of the potatoes, it was found that under different fertilization treatments, the potato plant height (PH), aboveground dry weight (DW), and SPAD values were highly significantly positively correlated with the yield, single-plant weight (PW), number of tubers per plant (TN), number of marketable tubers per plant (MTN), and marketable tuber weight per plant (MTW). The net photosynthetic rate (Pn) was significantly correlated (*p* < 0.05) with the transpiration rate (Tr), stomatal conductance (Gs), marketable tuber weight per plant (MTW), and the marketable tuber number per plant (MTN), and it was negatively correlated (*p* < 0.05) with the intercellular CO_2_ concentration (Ci). The Tr and Gs showed similar results to the Pn. The Ci was significantly negatively correlated (*p* < 0.05) with the single-plant weight (PW), number of tubers per plant (TN), marketable tuber weight per plant (MTW) and the marketable tuber number per plant (MTN). Furthermore, the yield was significantly positively correlated (*p* < 0.01) with the single-plant weight (PW), number of tubers per plant (TN), marketable tuber weight per plant (MTW), and number of marketable tubers per plant (MTN).

As shown in Table 3, principal component analysis of the potato growth, yield, and yield components was performed, and a comprehensive evaluation function was used to calculate the composite score. The results, shown in Table 3, indicated that the top three treatments were F2, F3, and F1, while CK ranked last. This suggests that under the experimental conditions, the F2 treatment is the most effective for promoting plant growth and increasing potato yield, making F2 the optimal formulation for the potato-specific water-soluble fertilizer.

### 2.6. Demonstration of the Optimal Fertilizer Formulation for Potato

As shown in Table 4, the results indicate that the single tuber number per plant, single-tuber weight, single-plant yield, marketable tuber rate, and yield per acre of potatoes were all significantly better than those under conventional fertilization. Compared to the CK, these parameters increased by 13.46%, 1.47%, 14.78%, 8.31%, and 14.79%, respectively. This suggests that the potato-specific fertilizer F2 significantly improves tuber quality and increases potato yield.

**Figure 5:**
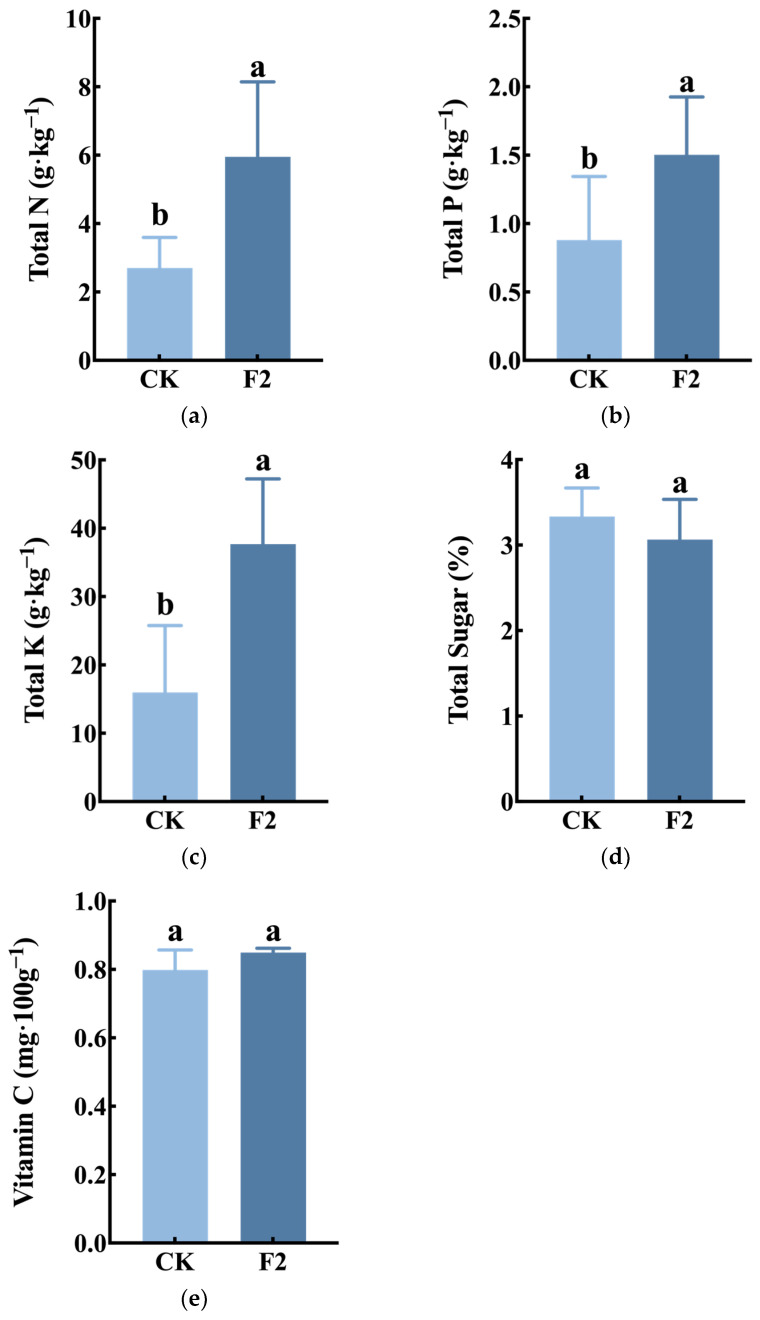
Effect of F2 fertilization on potato quality. (**a**): Total N. (**b**): Total P. (**c**): Total K. (**d**): Total sugar. (**e**): Vitamin C. The error bars represent the standard error of the mean (SEM). Statistical significance was determined using an independent sample *t*-test, different lowercase letters indicate significant differences among treatments (*p* < 0.05).

Figure 5a–c show that the F2 treatment significantly increased the accumulation of total nitrogen, total phosphorus, and total potassium in the tubers, with increases of 120%, 71%, and 136%, respectively, compared to the CK. This demonstrates that the potato-specific fertilizer F2 optimizes nutrient accumulation and improves tuber quality. However, there was no significant difference in the increase of total sugar and vitamin C content in the tubers.

## 3. Discussion

The advantage of the new fertilizer is its ability to reduce fertilizer use while enhancing efficiency. In this study, based on the fertilization requirements of potatoes, a potato-specific formula fertilizer was developed by using smaller amounts of nitrogen, phosphorus, and potassium fertilizers through chelation. This approach successfully achieved improvements in both the quality and yield of potatoes. The use of this specialized fertilizer enabled the enhancement of nutrient efficiency and optimized the overall growth and productivity of the crop while reducing the environmental and economic costs associated with excessive fertilizer application.

Photosynthesis is fundamental to crop growth, development, yield, and quality formation, with approximately 90% of crop yield stemming from photosynthesis. This study shows that, compared to the control group (CK), the net photosynthetic rate (Pn), transpiration rate (Tr), and intercellular carbon dioxide concentration (Ci) of the fertilization treatments (F1, F2, and F3) did not differ significantly (Figure 2a,b,d). However, all treatments exhibited a consistent physiological response trend: the Pn and Tr increased by 32.22–56.23% and 27.06–46.20%, respectively, while the Ci decreased by 11.97–29.98%. These results align with the findings of Zhang et al. [26]. No significant differences were found in the Pn, Tr, and Ci among the fertilization treatments, likely due to factors such as high-temperature, high-light environments, low soil phosphorus availability, and the measurement timing. Notably, the stomatal conductance (Gs) in the F2 treatment was significantly higher than in the CK. Given the largest yield increase observed in the F2 (121.29% higher than the CK), it can be inferred that this improvement is due to the dual effect of fulvic acid and the balanced N-P-K ratio in the F2 formulation. This treatment likely optimizes root activity and stomatal regulation, enhancing gas exchange rates [27]. The Pn, Tr, and Gs of the F3 treatment did not show significant advantages. It is speculated that the relatively low concentration of fulvic acid (30 g·L^−1^) has no positive effect on the photosynthesis of potatoes [28,29]. In conclusion, the yield-enhancing mechanism of the fertilization treatments is not solely dependent on increased photosynthetic rates but rather achieved through the synergistic optimization of the stomatal conductance (Gs) and nutrient use efficiency. The “fulvic acid-balanced nutrition” coupling mode of the F2 treatment significantly improved the Gs and yield, providing a theoretical foundation for the precise fertilization strategy of “adjusting fertilizer with water and promoting photosynthesis via stomata” for crops in arid regions [30].

The experimental results indicate that during the tuber enlargement stage, the plant height, stem diameter, aboveground dry matter, and relative chlorophyll content of potatoes followed the following order: F2 > F3 > F1 > CF > CK. Among them, the F2 treatment significantly increased the plant height and dry matter by 24.43%, 55.71%, respectively, compared to the CF treatment. Although no significant difference in relative chlorophyll content is observed between the F2 treatment and the CF treatment, the relative chlorophyll content in the F2 treatment is significantly higher than in the CK control group, with an increase of 12.96%. This demonstrates that applying a specialized water-soluble fertilizer for potatoes can significantly promote plant height, dry matter accumulation, and chlorophyll content in potatoes. These findings are consistent with the research of Guo Yuxin [31], which showed that the combination of humic acid with chemical fertilizers increased potato plant height by 3.18–10.76% and stem diameter by 4.96–13.22%. Yuan et al. [32] also showed that the application of humic acid improved the potato plant height, chlorophyll content, and dry matter accumulation, enhancing physiological characteristics. Ali et al. [17] stated that the application of humic substances improves water and nutrient supply to plants, promotes the growth of both potato plants and tubers, and enhances nutrient use efficiency, leading to better plant growth and higher dry matter yield [18,33]. These studies suggest that humic acid not only regulates plant growth but also increases crop yield and improves crop quality. However, during the seedling stage, the CF treatment showed the highest plant height, while the humic acid-containing specialized water-soluble fertilizer exhibited noticeable effects later in growth. It is speculated that during the seedling stage, fertilizers primarily influence potato plant height and root development. The application of appropriate nitrogen, phosphorus, and potassium promotes early-stage growth, while humic acid has a more limited impact on seedling growth and mainly enhances later-stage growth and stress resistance [34,35,36]. The application of specialized water-soluble fertilizers for potatoes resulted in higher yield, better composition elements, and improved economic benefits compared to the control group. This indicates that the specialized fertilizer can significantly increase potato yield and thereby enhance economic benefits. Previous studies have shown that combining humic substances with fertilizers significantly increased tuber yield by 9.3% compared to using fertilizers alone and by 2.9% compared to applying humic substances alone [18,33]. The use of organic–inorganic mixed fertilizers containing humic acid significantly improved economic traits and increased yield and benefits, resulting in yield increases of 9.28% and 4.6%, respectively [37]. These results are consistent with the findings of this study. Similarly, crops like maize and bamboo shoots treated with humic substances have shown similar yield increases [38,39,40]. This could be due to humic substances enhancing plant photosynthesis and respiration rates, increasing plant hormone-like activity, reducing nitrogen and potassium leaching, raising the content of available phosphorus in deep soil, and improving the plant growth environment, thus promoting crop development [41,42,43,44,45,46,47]. Additionally, humic substances help form soil aggregates, protect potato tubers from soil coverage during all growth stages, and improve tuber quality. Fulvic acid substances also improve soil water retention and nutrient supply capacity, enhancing root growth [21].

In terms of fertilizer use efficiency, the specialized water-soluble fertilizer for potatoes showed the following trend in fertilizer partial productivity and agronomic fertilizer use efficiency: F2 > F3 > F1 > CF. Among them, the F2 treatment exhibited the highest fertilizer partial productivity and agronomic fertilizer use efficiency, increasing by 90.67% and 166.5%, respectively, compared to the CF treatment. This study, based on the actual fertilization practices of local farmers (CF), investigates feasible solutions for achieving both stable yields and enhanced efficiency. The nitrogen application in conventional fertilization (CF) (258 kg·ha^−1^) exceeds the internationally recommended range (160–200 kg·ha^−1^), while the nitrogen levels in F1 (174 kg·ha^−1^), F2 (185 kg·ha^−1^), and F3 (208 kg·ha^−1^) were reduced by 32.56%, 28.29%, and 19.38%, respectively, compared to the CF. The results show that F2, with a 28.29% reduction in nitrogen fertilizer, significantly increases fertilizer productivity without a notable decrease in yield in contrast to the CF. This is likely due to the moderate fulvic acid content (60 g·L^−1^) in the F2 treatment, which enhances nutrient absorption while avoiding the root inhibitory effects associated with higher fulvic acid concentrations, such as in F1 (120 g·L^−1^) [48,49]. Additionally, F2’s balanced N-P-K ratio supports the crop’s diverse nutritional needs at different growth stages, thus optimizing fertilizer use efficiency [50,51]. This suggests that, even without reaching the international nitrogen threshold, substantial reductions in fertilizer use and improvements in efficiency are achievable by refining fertilizer formulas (e.g., incorporating fulvic acid and adjusting N-P-K ratios) based on existing farmer practices. However, while the F1 treatment reduced nitrogen to the international recommended threshold (174 kg·ha^−1^), its yield increased by 6.52% (*p* < 0.05) compared to the CF, and its fertilizer use efficiency was the lowest, possibly due to the high fulvic acid concentration (120 g·L^−1^), which inhibits nutrient uptake by the roots [49]. Although the fertilizer use efficiency of the F3 treatment was lower than that of the F2 treatment, it was still significantly higher than the CF. The nitrogen rate (208 kg·ha^−1^) in the F3 treatment promoted early crop growth, boosting fertilizer productivity and agronomic efficiency [52]. Nevertheless, the lower fulvic acid content (30 g·L^−1^) in the F3 treatment resulted in a weaker effect on nutrient absorption and utilization [53,54]. In conclusion, this study defines an optimal nitrogen reduction range for potato cultivation (20–30%) and offers farmers a practical transitional approach that balances reduced fertilizer input with stable yields. The findings demonstrate that optimizing fertilizer formulations can significantly enhance fertilizer use efficiency, and further research is needed to explore the potential of specialized fertilizer mixes at lower nitrogen levels.

Additionally, this study was conducted in an arid region of northwest China, where the soil pH is 8.21. Alkaline soils can limit the availability of certain nutrients, such as phosphorus and micronutrients. However, through adaptive management practices, successful crop cultivation has been achieved in alkaline soils within the pH range of 7.5–8.5 in the arid regions of Xinjiang, Shaanxi, and Gansu [55,56,57,58]. This experiment confirms that the application of a specialized fertilizer formula containing fulvic acid can increase yield and improve fertilizer use efficiency. This effect may stem from the synergistic action of fulvic acid and a balanced N-P-K ratio, which promotes high yield and quality tuber production. Fulvic acid, as an organic active substance, enhances the availability of phosphorus and micronutrients through its strong chelation capacity, improves soil structure, and facilitates nutrient uptake by the roots [35]. Studies have shown that fulvic acid can chelate with minerals such as iron, calcium, copper, zinc, and magnesium, directly delivering these elements to the plant, thus enhancing the absorption of micronutrients [21,23]. Therefore, despite the high soil pH in the study area, effective fertilization management successfully mitigated the negative impact of alkaline soils on potato growth, significantly increasing both yield and fertilizer use efficiency. In addition, it should be noted that the wide row spacing model employed in this experiment is primarily tailored for intensive agricultural production in the northwest. When applied to other regions, adjustments should be made according to local agronomic practices. Future studies should further investigate the interaction mechanisms between planting patterns and fertilizer efficiency through comparative experiments involving multiple row spacing configurations.

## 4. Materials and Methods

### 4.1. Overview of the Experimental Area

The experiment was conducted on 16 May 2023 at the Baicheng Agricultural Experiment Station, Kangqi Township, Baicheng County, Aksu, Xinjiang (41°47′ N, 81°54′ E). The region has a temperate continental arid climate characterized by cold winters and cool summers. The experimental station is located at an altitude of 1178.4 m, with an average temperature of 22.18 °C during the study period, total precipitation of 43.2 mm, and a monthly average humidity of 53.06%. Climate data were obtained from the Baicheng Meteorological Station.

Before the experiment, soil samples were collected from four points at a depth of 0 to 20 cm. After homogenization, the samples were analyzed for their chemical and physical properties. The soil used in the experiment was loam, with its basic physical and chemical properties shown in Table 5.

### 4.2. Experimental Design

**Figure 6:**
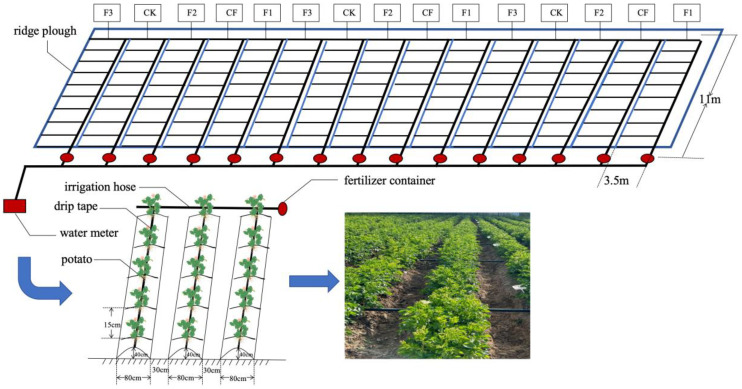
Potato cultivation model.

The experiment was conducted using the potato variety ‘Xisen 6′ with five treatments: (1) control group (CK: no fertilizer applied); (2) conventional fertilization (CF: N-P-K = 258-245-338 kg·ha^−1^); (3) formulated fertilizer 1 (F1: 120 g·L^−1^ fulvic acid; N-P-K = 110-100-120 g·L^−1^); (4) formulated fertilizer 2 (F2: 60 g·L^−1^ fulvic acid; N-P-K = 130-120-150 g·L^−1^); and (5) formulated fertilizer 3 (F3: 30 g·L^−1^ fulvic acid; N-P-K = 170-150-130 g·L^−1^). A completely randomized design was adopted, with each treatment replicated three times, resulting in a total of 15 plots. Each plot covered an area of 38.5 m^2^ (11 m × 3.5 m), as shown in Figure 6.

A drip irrigation volume of 3705 m^3^·ha^−1^ was applied to all five treatments. The potato variety used in the study was ‘Xisen 6’, which was developed through a sexual hybridization system by the National Potato Engineering Technology Research Center and Laoling Xisen Potato Industry Group Co., Ltd. (Laoling, China) by crossing ‘Shepody’ as the female parent and ‘XS9304’ as the male parent [59]. This variety exhibits strong adaptability, high yield, superior quality, disease resistance, and tolerance to saline–alkaline conditions [60,61,62].

Potatoes were sown on 16 May 2023. Before planting potatoes, urea (46.4% N) at 75 kg·ha^−1^, diammonium phosphate (18% N, 46% P) at 210 kg·ha^−1^, and potassium sulfate (50% K) at 225 kg·ha^−1^ were applied as the base fertilizer. During the entire growing season (seedling, flowering, tuber bulking, and starch accumulation stages), the CF treatment received a total of 240 kg·ha^−1^ of urea, 240 kg·ha^−1^ of diammonium phosphate, 375 kg·ha^−1^ of potassium sulfate, and 225 kg·ha^−1^ of compound fertilizer (17% N, 17% P, 17% K). Each formulated fertilizer treatment received a total of 75 kg·ha^−1^ of urea, 15 kg·ha^−1^ of diammonium phosphate, 75 kg·ha^−1^ of potassium sulfate, and 750 kg·ha^−1^ of formulated fertilizer. The total fertilizer amounts for each treatment are summarized in Table 6. Potatoes were planted using a single ridge and single row planting method. The ridge height was 40 cm, the ridge width was 80 cm, and the furrow width was 30 cm. The potato plant spacing was 15 cm, with a sowing depth of 30 cm, resulting in a planting density of 4600 plants per acre. Each plot contained a fertilizer tank, and drip irrigation lines were laid on the ridge surface to apply fertilizer through drip fertigation. Normal field management practices were followed during the trial, and the potatoes were harvested on 31 August. In 2024, we conducted field demonstrations to promote the optimal potato-specific fertilizer formula selected from our 2023 field experiments.

### 4.3. Measurement Indicators and Methods

#### 4.3.1. Plant Growth

During the seedling stage, bud stage, tuber bulking stage, and starch accumulation stage of potato growth, three randomly selected plants from each treatment in each plot were measured for plant height and stem diameter. The average values for each plot were taken to represent the overall growth of the plants in that plot. Plant height was measured using a steel tape measure with an accuracy of 1 cm, and stem diameter was measured using a vernier caliper at the point where the stem contacts the soil, with an accuracy of 0.1 mm.

#### 4.3.2. Plant Physiological Indicators Measurement

On a clear morning during the tuber bulking stage, the relative chlorophyll content (SPAD value) was measured using a SPAD meter. The largest leaf of each plant was selected for measurement, and three points on each leaf were measured to obtain an average value.

#### 4.3.3. Aboveground Dry Matter Accumulation Measurement

During the seedling stage, bud stage, tuber bulking stage, and starch accumulation stage of potatoes, three plants from each treatment in each plot were selected. After removing surface dirt, the plants were placed in an oven at 105 °C for 30 min to blanch and then dried at 75 °C until reaching a constant weight. The aboveground dry matter accumulation was measured using a balance, and the average value was calculated for each plot [63].

#### 4.3.4. Photosynthetic Characteristics Measurement

During the tuber bulking stage on 20 July, between 9:00 and 10:00 a.m., three plants from each plot were randomly selected. For each plant, three healthy leaves from the same leaf position were chosen. The net photosynthetic rate (Pn), transpiration rate (Tr), stomatal conductance (Gs), and intercellular CO_2_ concentration (Ci) were measured using a LI-6400 portable photosynthesis system (LI-COR Inc., Lincoln, NE, USA). The average values for each plot were calculated to represent the photosynthetic data during the bulking stage.

#### 4.3.5. Potato Yield and Yield Components Measurement

After potato maturity, all potatoes in each plot were harvested and weighed to determine the total yield, which was then converted into standardized yield. Additionally, three randomly selected plants from each plot were weighed to determine the weight of individual tubers, the weight of tubers per plant, and the number of tubers per plant. The number of marketable potatoes (tubers weighing over 75 g) and marketable tuber weight per plant were also recorded, and the marketable rate and economic benefit were calculated. Subsequently, three randomly selected potato tubers were placed in sealed bags and taken to the laboratory for quality analysis.

#### 4.3.6. Calculation of Fertilizer Partial Factor Productivity (PFP, kg·kg^−1^)

The formula for PFP is as follows:

PFP = Y/F_T_

where Y = Crop yield (kg·hm^−2^); F_T_ = Total amount of fertilizer applied during the entire growing season (kg·hm^−2^) [55].

#### 4.3.7. Calculation of Agronomy Fertilizer Use Efficiency (AFUE, kg·kg^−1^)

The formula for AFUE is as follows:

AFUE = (Y − Y_0_) /F_T_

where Y = Crop yield with fertilizer applied (kg·hm^−2^), Y_0_ = Yield from the control treatment (no fertilizer applied) (kg·hm^−2^), F_T_ = Total amount of fertilizer applied during the entire growing season (kg·hm^−2^).

#### 4.3.8. Quality Measurement

Total sugar content was determined using the phenol-sulfuric acid colorimetric method. Vitamin C content was measured using the molybdenum blue colorimetric method [64].

### 4.4. Statistical Analysis

The data were processed using Microsoft Excel 2021, and statistical analysis was performed with IBM SPSS Statistics 28. One-way analysis of variance (ANOVA) is employed to evaluate the differences between field trial groups. When significant differences are identified, post-hoc multiple comparisons are conducted using the Tukey HSD test. An independent samples *t*-test is utilized to assess the significance of the mean differences between the field demonstration experimental group and the control group. Graphs were generated using Origin 2021. Pearson’s method was used for correlation analysis, and a correlation heatmap was created using ChiPlot (https://www.chiplot.online/; accessed on 10 December 2024).

## 5. Conclusions

This study, based on a one-year field trial, provides preliminary evidence that the novel water-soluble fertilizer F2 (60 g·L^−1^ fulvic acid, N-P-K = 130-120-150 g·L^−1^) can significantly improve the agronomic traits and photosynthetic performance of potato cultivar “Xisen 6”. The enhancement of stomatal conductance (Gs) and relative chlorophyll content (SPAD value) facilitated the accumulation of dry matter in the aboveground parts and the enrichment of tuber nutrients, with nitrogen, phosphorus, and potassium contents increasing by 120%, 71%, and 136%, respectively. This led to an increase in both yield and economic efficiency, with the F2 treatment yielding 34.6% more than conventional fertilization and increasing economic benefits by 23,523.1 RMB·ha^−1^ under specific wide row conditions. Although the effects of wide row spacing and high light intensity may have amplified the root-promoting effects and photosynthetic potential of the F2 treatment, the short duration of the one-year trial and the specificity of the cultivation mode limit the generalizability of the results. Therefore, the long-term stability and adaptability to different planting systems require further validation through multirow comparisons and long-term trials. The results of this study suggest that the F2 fertilizer formula has significant potential for improving fertilizer efficiency in potato production in arid regions. However, its large-scale promotion should be based on local agronomic practices to optimize fertilization strategies, thus promoting the standardized application of green, high-yield technologies.

## Figures and Tables

**Table 1:** Economic efficiency analysis of potato yield under different fertilizer treatments.

Treatment	Yield (t·ha^−1^)	Total Revenue (RMB·ha^−1^)	Cost (RMB·ha^−1^)	Net Income (RMB·ha^−1^)	Increase in Income Relative to CF (RMB·ha^−1^)	Production-to-Investment Ratio
CK	26.9 ± 3.5c	40,326.9 ± 5292.7c	36,750.0	3576.9 ± 5292.7d	−21,407.9 ± 9349.8b	1.1 ± 0.1c
CF	44.2 ± 2.9b	66,302.3 ± 4267.5b	41,317.5	24,984.8 ± 4267.5c	-	1.6 ± 0.1b
F1	47.1 ± 0.7b	70,625.8 ± 1044.9b	40,732.5	29,893.3 ± 1044.9bc	4908.4 ± 4952.2a	1.7 ± 0.3b
F2	59.5 ± 2.9a	89,241.4 ± 4325.1a	40,732.5	48,508.9 ± 4325.1a	23,523.1 ± 2063.0a	2.2 ± 0.1a
F3	55.6 ± 1.4b	83,344.6 ± 2161.3ab	40,732.5	42,612.1 ± 2161.3ab	17,627.3 ± 3281.6a	2.1 ± 0.1ab

Data are mean ± standard error of the mean (SEM). Different lowercase letters indicate significant differences among treatments (Tukey test, *p* < 0.05).

**Table 2:** Effects of different fertilization treatments on fertilizer use efficiency in potato.

Treatment	PFP (kg·kg^−1^)	AFUE (kg·kg^−1^)
CK	-	-
CF	52.5 ± 3.4b	20.6 ± 7.4a
F1	86.1 ± 1.3a	36.9 ± 6.3a
F2	100.1 ± 4.9a	54.9 ± 9.7a
F3	89.2 ± 2.3a	46.0 ± 7.8a

Data are mean ± standard error of the mean (SEM). Different lowercase letters indicate significant differences among treatments (Tukey test, *p* < 0.05).

**Table 3:** Comprehensive evaluation of potato growth and yield components under different fertilization treatments based on principal component analysis.

Treatment	Composite Score	Rank
CK	−2.83	5
CF	−0.66	4
F1	0.27	3
F2	1.95	1
F3	1.28	2

**Table 4:** Potato yield and its components under the F2 fertilization treatment.

Treatment	Tuber Number (tuber·plant^−1^)	Single-Tuber Weight (g·tuber^−1^)	Plant Weight (g·plant^−1^)	Marketable Rate (%)	Yield (t·ha^−1^)
F2	5.9 ± 0.23a	212.58 ± 10.08a	1235.83 ± 29.90a	93.82 ± 0.01a	72.48 ± 1.75a
CK	5.2 ± 0.20b	209.51 ± 7.99a	1076.67 ± 20.99b	86.62 ± 0.01b	63.14 ± 1.23b

Data are mean ± standard error of the mean (SEM). Statistical significance was determined using an independent sample *t*-test, different lowercase letters indicate significant differences among treatments (*p* < 0.05).

**Table 5:** Basic physical and chemical properties of the soil at the experimental site in 2023.

Soil Depth (m)	Water-Soluble Salts (g·kg^−1^)	Water-Soluble Nitrogen (mg·kg^−1^)	Organic Matter (g·kg^−1^)	Available Phosphorus (mg·kg^−1^)	Total Nitrogen (g·kg^−1^)	Total Phosphorus (g·kg^−1^)	Total Potassium (g·kg^−1^)	pH
0~0.2	3.4	109.9	22	49.9	1.25	1.53	11.1	8.21

Soil analysis was performed at the Institute of Agricultural Resources and Environment, Xinjiang Academy of Agricultural Sciences.

**Table 6:** Fertilization scheme for potato fertilizer.

Treatment	Fulvic Acid (g·L^−1^)	N (kg·ha^−1^)	P (kg·ha^−1^)	K (kg·ha^−1^)
CK	0	0	0	0
CF	0	258.5	245.3	338.3
F1	100	173.6	161.1	212.2
F2	60	185.1	172.7	236.6
F3	30	208.2	190.1	225.0

## Data Availability

All data generated or analyzed during this study are included in this article.

## Abbreviations

The following abbreviations are used in this manuscript:

PH	Plant height
SD	Stem diameter
DW	Aboveground dry weight
SPAD	Relative chlorophyll content
Pn	Net photosynthetic rate
Tr	Transpiration rate
Gs	Stomatal conductance
Ci	Intercellular carbon dioxide concentration
PFP	Fertilizer partial factor productivity
AFUE	Agronomy fertilizer use efficiency
PW	Plant weight
TN	Tuber number
MTW	Marketable tuber weight
MTN	Marketable tuber number
